# Congestive Fever

**Published:** 1867-06

**Authors:** James C. Harris

**Affiliations:** Wetumpka, Alabama


*Congestive Fever.* By James C. Harris, M. D., Wetumpka, Alabama.

Before proceeding more particularly with the consideration of congestive fever, I beg leave to make the following explanatory remarks. In a communication entitled, "An Enquiry into the Nature and Existence of Typhoid Fever in the South," contained in the *New Orleans Medical and Surgical Journal*, Vol. 6 May No., 1850, page 712,* I used the following language: "Regardless, therefore of the objections that may be urged against the general application of the term *congestive* to the different varieties of malarial fever, those most familiar with the history of their symptoms will, we apprehend, readily admit that there is scarcely any grade, no matter how light, but that either at its commencement or some time during its progress, gives unmistakable signs of a greater or less determination of blood to some particular tissue or organ than another; that is broken balance of the circulation, continuing or recurring at regular or irregular intervals, is known by those who have studied the phenomena of congestion to produce, or be accompanied with, either increased or decreased nervous action, attended in the part to which the accumulation takes place with *swelling, pain, discoloration and heat;* that, coincident with these, the essential elements of inflammation, we have also increased, diminished, altered or suspended secretion, attended with the softening of the mucous membrane, effusion and ulceration; and that these within themselves are sufficient, and in our opinion do clearly indicate the nature and the name by which they should be known. To show that I am not singular in this opinon, and that others have been in the habit of making similar admissions, and accounting in the same way for some of the symptoms and *post-mortem* appearances, particularly in remittent fever, it is only necessary for me to direct the attention of my readers to the recorded views of Mr. Twining, who observes (*Diseases of Bengal*, chap. 5) that from the closest attention to clinical observations, as well as the result of *post-mortem* examination, he

*In an interesting article in the *Memphis Medical and Surgical Monthly*, for June, 1866, entitled "Observations on Epidemic and Endemic Diseases," by Lansford P. Yandell, M. D., we are informed (page 211—212) that typhoid fever in those portion of Tennessee with which he is familiar, and where it heretofore prevailed, has within the last few years entirely subsided.

is convinced that the remittent fevers of Bengal are invariably connected with local congestion, which often runs rapidly into inflammation, attended with much interstitial effusion. The seat of these local affections was found principally in the stomach, intestines, cellular structure about the duodenum, and at the root of the meso-colon, more especially where it passes across the spine; the principle disease being also often found in the spleen, liver, brain and lungs. Thus most clearly is shown, both from *post-mortem* facts and clinical observations, that while the mucous membrane of the intestinal tube, in the remittent fever of Bengal, is frequently the seat of inflammation and effusion, all the other organs of the body may in time take on a similar condition, and become *foci* of diseased action. Hence we would say arises the impropriety, from the occasional appearance of one *post-mortem* lesion (ulceration for instance of the glands of Peyer), of taking from a series of symptoms indicating and bearing the name of a clearly defined and well understood form of malarial fever, and hypothecating thereon a name, the very mention of which is calculated to mislead the practitioner, at least so far as treatment is concerned, into the most dangerous, not to say fatal, errors imaginable."

Again, in an essay on the climate and fevers of the South-Western, Southern Atlantic and Gulf States, reprinted from the Oct. and Dec. Numbers (1858)* of the *New Orleans News and Hospital Gazette*, I spoke of typhoid fever under the head of the *continued stage of remittent fever;* and in another unpublished part of the same Essay, of congestive fever, as the *congestive stage of intermittent and remittent fevers.* At the time of the appearance of the above essay, I contemplated, at no very distant day, the publication of a small volume upon the same subjects; and believed that this might contain some nosological reforms, tending to the introduction of a more rational and successful plan of treating typhoid and congestive fevers, particularly the latter, that ought to be promulgated. Recent circumstances rendering it uncertain whether I shall be able to effect this purpose, I have been induced on the present occasion (incidentally however), to present for the consideration of my professional brethren certain of these supposed reforms.

After a long and careful study of the causes, seats, symp-

---

* This Essay the editor of *De Bow's Review* for 1859 republished entire, in the June and July Numbers of that sterling periodical.

toms, anatomical characters and treatment of congestive fever, I have been brought to the conclusion that probably the chief cause of the disagreement in opinion amongst medical writers upon the subject, is perhaps more the result of an appropriate phrase to express the true pathological condition present in the congestive stage of intermittent or remittent fever than anything else. As we all know there is present more or less congestion in every grade and variety of ague, and that it is the most prominent, if not the most dangerous symptom in the cold stage of an intermittent that terminates in death during the first, second or any other paroxysm, we are unable to perceive any good reason why the epithet congestive might not be very properly applied to that condition of the system recognized and known amongst us by the familiar term of *congestive chill.* If the minds of our medical brethren could be brought fully to realize the truth, that a congestive chill is nothing more or less than a prolongation and perhaps deepening of the cold stage of the paroxysm of an intermittent, and that remittents during their course sometimes fall into a stage, I think they would experience little trouble in arriving at a correct understanding of the true nature of congestive fever.

In an essay on the distinctive character of congestive fever, Dr. Silas Ames* denies its identity with the pernicious fever of Dr. Wood, on account of the want of uniformity in their *post-mortem* lesions; and with the malignant intermittents and remittents of other writers, upon the ground that the latter preserve throughout their paroxysms the several (cold, hot and sweating) stages of a simple intermittent. Entertaining, as I do, the highest respect for the character and memory of Dr. Ames, it is really a source of no small regret to have to differ with him upon any subject, but more especially upon one in which his experience could not have been otherwise than ample; nevertheless I am, at least for the present, unwilling to admit that the absence of inflammation in any of the tissues or organs of the bodies of those who die in the first or second chill, or the presence during life of some symptoms in the paroxysm of an intermittent or remittent fever that are absent in others, proves any non-identity whatever between them and congestive fever.

---

* New Orleans Medical and Surgical Journal, Vol, Nov., 1850, page 300.

Under the head of congestive fever, Dr. S. H. Dickson,† while refusing to admit there is any variety of fevers to which this title is exclusively appropriate (in compliance with what he considers custom among American writers, who thus designate a particular), describes a periodical form of intermittent and remittent fever. It is chiefly the latter variety which he thinks, if not identical with, at least to resemble very much the malignant intermittents and remittents of the French and Italian writers. Again, Dr. Drake‡, after describing simple and inflammatory intermittent fever, then comprehends the remaining varieties of this type, not referable to these two heads, in which the reäction and remission are feeble and imperfect, and the regularity of the other symptoms mingled or wanting under that of malignant intermittent. He also further informs us (page 114—115) that in malignant remittent fever, there is not only present great congestion in the vena cava and its branches, but there also exists a broken balance in the circulation, and that the organs most oppressed become the seats of special irritation and congestion. When these local inflammatory engorgements fail to produce reäction, as they sometimes do, we have present every element of danger and difficulty, together with a concourse of symptoms of a highly adynamic and ataxic character, from which the patient will be recovered with difficulty.

In some localities in the Southern and South-Western States, those in which during the summer and fall months malarial fevers of every grade and variety prevail, we occasionally meet, in the second or third paroxysm of remittent fever, with a weak, frequent and variable pulse, attended with increased gastric irritability, restlessness and thirst. These symptoms, generally denominated by medical writers malignant or pernicious, are really nothing more than the premonitory signs of an approaching cold stage, and the manifest result alone of congestion. In other cases the congestion falls more particularly on some one or more of the principle organs of the body, as the brain, lungs, liver, spleen, stomach or bowels, aud is accompanied in every instance by the peculiar symptoms characteristic of hyperæmia of these organs. These local determinations du-

† Elements of Medicine, pages 258—9.

‡ Principle Diseases, Int. Val. N. America, Vol. 2, page 71.

ring the course of the primary fever have been, I think, very erroneously described by medical writers as the comatose, soporose, thoracic and abdominal varieties of congestive fever. In other low, moist localities, where the long absorption of malaria has destroyed the vitality of the blood, turned it black and rendered it incapable of stimulating the heart into reäction, the congestive stage is neither so mild or gradual in its approach, the patient frequently dying in the first chill. These necræmial cases are very well described by Dr. Lewis, in his "Medical History of Alabama;" by Dr. Hart, * of New Orleans, and others. We are informed by Dr. Forry, † that they are ushered in with a prolonged sense of cold and universal collapse of the vital powers, occurring in places and at times in which the endemic causes are intense and concentrated; but as they are of rather rare occurrence and almost universally fatal, they cannot, I think, be viewed with propriety in any other light than as aggravated cases of the congestive stage, in which the power of reäction is completely overwhelmed.

From an examination of the preceding synoptical extracts, it appears that, while some of the writers cited contend for a congestive fever, *abnitio*, others no less experienced deny its existence altogether, or describe a similar condition of the system under the name of malignant, intermittent, remittent, or pernicious fever. The want of respect, on the part of one of these writers, ‡ for the congestive theory, the advocates of which he derisively styles hydraulic or mechanical pathologists, no less than the difficulty experienced by some of the others in the transformation of a *mere symptom* into an idiopathic disease, is probably to some extent, the cause of difference amongst them. Hence the classification, by systematic writers upon congestive fevers, into the cerebral, thoracic and abdominal forms, with sometimes a subdivision of these into varieties. I feel constrained to maintain, upon the grounds just stated, that the original disease of which they are mere species, varieties and subdivisions, is itself nothing more than a symptom, a stage of periodic fever, and should be described and treated as such.

*Treatment.*—To meet all the indications, this must be pre-

* "New Orleans Medical and Surgical Journal." Vol. IV. July, 1847, page 56.

† Forry's "Climate of the United States," page 287.

‡ "Drake's Principal Diseases, Interior Valley of N. America," Vol. II, page 114.

ventive in the chill and during the remission. In an attack of either intermittent or remittent fever, should any of the symptoms present themselves heretofore mentioned as premonitory of the approach of the congestive stage, we must endeavor to bring the system of our patient under the influence of quinine and opium, before the expected return of the next paroxysm. This may generally be affected by the administration, every two or three hours, of from four to six grains of quinine, and half a grain of opium, or its equivalent of morphine, until from 20 to 30 grains of the former are taken. Sometimes, in combination with each dose of quinine and opium, we give from 3 to 5 grains of pulverized cayenne pepper, directing the patient at the same time to drink pretty freely of a tea of this article, and to apply a mustard poultice to the epigastrium. This course I have generally found efficient to prevent the development of the congestive stage. Afterwards, if there is present much hepatic derangement, which is frequently the case, I give some three or four pills (one every two hours), composed of two or three grains each of calomel and blue-mass, to be followed, if necessary, with oil; and then complete the cure by giving, for two or three days longer, more quinine, gradually reducing the quantity each day.

Dr. Horatio N. Morris and myself, in the fall of the year 1835, in the case of a young gentleman at the "Planters' Hotel" in this city (Wetumpka), in the congestive stage of an intermittent fever, gave him, during the course of the afternoon, some eighty or ninety grains of quinine, with as much brandy and opium as we thought prudent; at the same time applying mustard poultices to his spine, epigastrium and extremities. Yet our patient gradually grew colder and weaker, and finally, before midnight expired. This case, together with several others that came under my observation within the next five or six years, in which large quantities of quinine, aided by the external application and internal administration of the most powerful stimulants, failed tò produce the least reäction, led me, not only to doubt the curative powers of eighty or one hundred grains of quinine in this particular condition of the system, but to suspect this quantity might have had something to do in bringing about the fatal result. About this time, and when greatly discouraged in trying to fix upon some better plan of treating the congestive stage of fever, I received from my friend Dr. W. O. Baldwin, of Montgomery, his essay on the poisonous properties of the sulphate of quinine. After a thorough study of this essay, a few rays of light began to illuminate the darkness by which I was surrounded, confirming my previous suspicions, and leading me irresistably to the conclusion that from twenty to thirty-six grains of pure quinine would produce that particular condition of the system recognized by us all as quininism, a state of irritation and excitement;* that carried beyond this point, it would begin to display its sedative powers, and perhaps a little further, its poisonous properties. Ever since this time, now some eighteen or nineteen years ago, I have never doubted that large doses of this remedy in stages of depression (no matter what may be its action in other conditions of the system), would produce dangerous symptoms, if not death; and consequently have discontinued its use in large doses, during the chill. The plan now pursued in the chill, is to apply immediately over the epigastric region a large blister, six by eight, or eight by ten inches, letting it remain on from eight to ten hours. If I have any fears, from the coldness and want of vitality in the skin, that the blister may not draw promptly, I endeavor to irritate the surface with mustard or the spts. of turpentine, taking care at the time of the application of the blister to moisten it well with the latter article. This done, I make use of the following:

℞—Quinine sulph., gr. xxxvi;
Calomel, gr. xviii;
Opii, gr. iv;
Camphor, gr. vi;
Olei piper. nig., gut. xviii;
Confection. ros., q. s. M;
Ft. pil., xviii.

One of these I give every two hours through the congestive stage, and continue them until the patient has passed beyond the period for the return of the next paroxysm; which will be in from twenty-four to thirty-six hours, according to the type (tertian or double tertian) of the prevailing fevers of the locality. During the remission, if the pills have not acted, give a little oil, or some other mild laxative; and then continue to treat the case, but rather carefully, as an ordinary intermittent or remittent fever.—*N. O. Med. and Surg. Jour.*

---

* "Dickson's Elements of Medicine," Revised Edition, page 262.

